# Segmental Dilatation of Near Total Colon Managed by Colon Preserving Surgery

**Published:** 2012-09-01

**Authors:** M Ragavan, S Arunkumar, NS Balaji

**Affiliations:** Dept. of Pediatric Surgery MIOT Hospital 4/112, Mount Poonamalle Road. Manapakkam Chennai.600089.

**Keywords:** Segmental dilatation, Colon, Colorraphy

## Abstract

Segmental dilatation of the colon is a rare congenital disorder of colonic motility and often involves a short segment that causes chronic constipation in children. There are only 10 cases of neonatal colonic segmental dilatation described in literature. We managed a case who presented in the neonatal period with abdominal distension. There was dilatation of whole of the colon except part of ascending colon and the rectum. The case was managed by tubularization of the segmental dilatation of colon with stoma formation as first stage followed by delayed anastomosis during second stage.

## INTRODUCTION

Segmental dilatation of colon [SDC] is a rare congenital entity characterized by a segmental dilatation of variable length, with the proximal and distal bowel of normal caliber. Clinical and radiological features resemble that of Hirschsprung’s disease and hence labeled under ‘disorders related to Hirschsprung’s disease [1,2]. It was first described by Swenson and Rathauser in 1959 [3]. These patients are managed by resecting the diseased segment and restoration of the continuity of the bowel by primary or delayed anastomosis. In this report, we present a case with near total SDC of colon managed by diversion stoma and colorraphy followed by restoration of continuity. 

## CASE REPORT

A 4-day-old newborn weighing 2.8kg, presented with abdominal distension, respiratory distress with non-passage of meconium. Examination showed grossly distended and resonant abdomen with dilated veins on the surface. Anal opening was normal and a 10 size red rubber catheter could be passed without difficulty. There was no visible bowel loops or palpable mass. Nasogastric tube drained no aspirate. Clinically a diagnosis of neonatal bowel perforation was made. X-ray abdomen showed large gas and fluid level with deviation of nasogastric tube to right side (Fig. 1A). There was no rectal gas shadow. Possibility of gastric perforation or meconium cyst with communication with bowel was kept in mind. Fluoroscopic water soluble contrast gastrography was done on table that showed normal stomach with flow of contrast to small bowel ruling out gastric perforation. Contrast enema was done to look for microcolon assuming that meconium cyst as a possibility. It showed enhancement of the gas filled structure (Fig. 1B). A diagnosis of either meconium cyst, duplication cyst with communication and contained perforation was considered. 

Laparotomy was done via right upper transverse incision. The stomach and small bowel were normal looking. The incision was extended across the midline to deliver a spherical shaped large dilated segment measuring 17 cm x15 cm involving most of the colon except for the normal sized proximal 4 cm of ascending colon and distal rectum from the level of S3 vertebrae (Fig. 2A, 2B). The appendix was single and normal. The blood supply for the dilated part was from ileocecal artery. The inferior mesenteric artery was found running along the mesenteric side as an arcade and branching dichotomously on either side, to supply the bowel wall. There were no creeping or serpentine blood vessels on the antimesenteric side as in pouch colon. Malrotation was present with cecum in the left hypochondrium and thick Ladd’s bands. 

**Figure:**
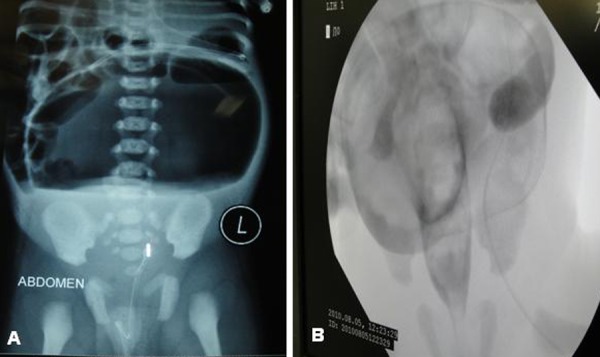
Figure 1: Plain X- ray abdomen showing large gas shadow with fluid level with deviation of nasogastric tube (A) and Contrast enema fluoroscopic image showing enhancement of gas filled structure (B).

**Figure:**
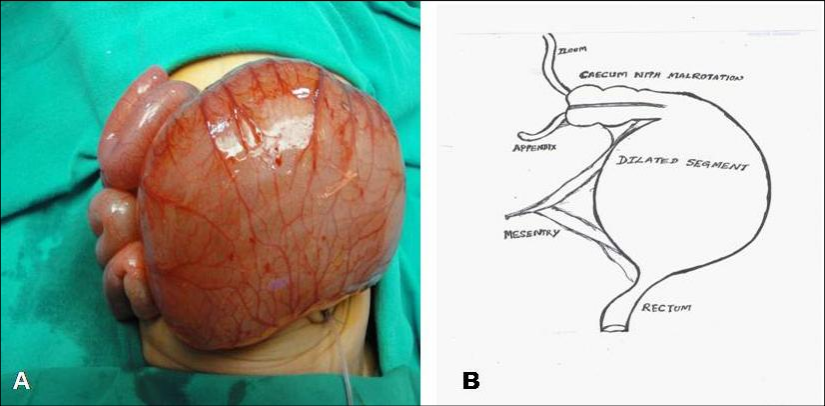
Figure 2: Per-operative picture showing near total segmental dilatation of colon (A) and the line diagram showing the extent of dilatation (B).

The colon was divided at the junction of ascending colon and dilated segment and proximal end colostomy was done. In view of near total colonic involvement, the dilated distal part was subjected to colorraphy and brought out as distal stoma. Ladd’s bands were divided and appendicectomy done. The excised part of the dilated colon and appendix were sent for biopsy which showed normal ganglion cells and muscle layer. Post operatively the proximal stoma functioned well. 

After 3 months, to assess the function of distal preserved colon, rectal biopsy, anorectal manometry and transit study were done. For transit study 10 capsules were loaded with barium into the distal stoma. X-ray abdomen was taken every 2 hour. All the capsules were expelled out of anus within 6 hours. Distal cologram showed normal caliber colon of adequate length with no retention of barium in 24 hours film. There was no recurrence of dilatation. The rectal biopsy and anorectal manometry were normal. Colostomy closure was then performed. In the post operative period baby passed liquid stool in 48 hours and then breast feeding was started. There was no constipation or other symptoms at one year follow-up. Repeat barium enema done at 6 months showed normal caliber colon.

## DISCUSSION

Brawner and Shafer summarized clinical and pathologic features of CSD of colon as:

- Lack of radiographically demonstrable motility of the dilated segment;
- Normal appearing and functioning colon both proximal and distal to the dilated segment;
- Absence of taenia coli in the dilated segment;
- Normal ganglion cells;
- Hypertrophy of the circular and longitudinal muscle layers in the dilated segment [2-5].

The left side of the colon is affected more frequently, with sigmoid and rectosigmoid involvement in 45% of cases [6]. In our case there was involvement of whole of colon except part of ascending colon and rectum. Cases involving the colon have a clinical picture very similar to that of Hirschsprung's disease and usually present with constipation in childhood or later (6 month to 22 year) [1,7]. Involvement of ileum or jejunum usually manifest as neonatal bowel obstruction. Neonatal presentation in SDC is rare and only 10 cases have been reported so far [5,7-9]. 

The recommended treatment is resection of the dilated segment and end-to-end anastomosis with/without proximal colostomy [3,10]. In our case the resection of SDC would mean total colectomy which is not desirable; moreover it would also result in operative difficulty as anastomosis of ascending colon and rectum in pelvis is considered difficult with high leakage rates. Therefore, we planned a staged reconstruction with colorraphy and colostomy in the first stage followed by restoration of the bowel continuity during second stage after ruling out Hirschsprung’s disease and other motility disorders. We opted this option based on our experience of colorraphy in congenital pouch colon where the total length of colon is short and colorraphy is a good option though many authors do not favor it on account of intractable constipation problems in the postoperative period [11]. There is also a case report of preservation of the dilated segment by plication [12]. The functional analysis of the preserved dilated segment with available investigation in our institute and good outcome after surgery support the possibility of bowel preserving surgery in segmental dilatation when warranted. Though many cases presented mention that the dilated segment has motility problem we feel that if motility is the issue then intestinal obstruction and presentation in neonatal period is inevitable. The dilated segment has normal ganglion cells with no hypertrophy of nerve bundle, muscle layer and hence motility should be present. The presence of hypertrophied muscle layer in affected segment in late presenting cases and absence in early presenting cases suggests that there is ongoing contraction in the affected part to tide over the chronic obstruction. 

There are many associated malformations reported in such cases [6]. In our case malrotation was found. Malrotation has been described in 3 other patients with SDC. The dilated segment of significant size would have been present in the early period of embryogenesis and thus preventing normal rotation and fixation of bowel. Presence of associated malrotation though explains that the dilated part would have been of significant size in-utero but those cases also present few days after birth and following passage of meconium. This adds to our view that the obstruction may have mechanical element due to accumulation of gas and feces in the dilated segment. If the dilated segment is nonfunctional then muscle hypertrophy and dilatation should be present in the proximal bowel contracting against the obstructed site as in Hirschsprung’s disease. Mahadevaiah et al described certain abnormalities in like hypertrophied muscularis propria, nerve plexus, and ganglion cells located within the circular layer rather than the normal myenteric location in SDC [9]. These findings are not made out in other studies and do not explain the normal motility after excision of the redundant part by colorraphy in our case or after plication in another reported case [12].

## Footnotes

**Source of Support:** Nil

**Conflict of Interest:** None declared

## References

[1] ( 1982). Helikson M, Shapiro M, Garfinkel DJ, Shermeta DW. Congenital segmental dilatation of the colon. J Pediatr Surg.

[2] ( 1972). Brawner J, Shafer AD. Segmental dilatation of the colon. J Pediatr Surg.

[3] ( 1990). Al Salem AH, Grant C. Segmental dilatation of the colon: report of a case and review of the literature. Dis Colon Rectum.

[4] ( 1971). De Lorimer AA, Benzian RS, Gooding CA. Segmental dilatation of colon. Am J Roentgenol Rad Ther Nucl Med.

[5] ( 2010). Aayed R. AlQahtani. Laparoscopic-assisted sigmoid resection for colonic ectasia in a neonate. J Pediatr Surg.

[6] ( 1994). Gopal S, Gangopadhyay A, Pandit S. Segmental dilatation of sigmoid colon. Pediatr Surg Int.

[7] ( 1984). Molina E, Hidalgo F, Fernández S, Casanova A, Martín Sanz L. Segmental dilatation of the ileum. An Esp Pediatr.

[8] ( 2012). Mirza B, Bux N. Multiple congenital segmental dilatations of colon: a case report. J Neonat Surg.

[9] ( 2011). Mahadevaiah SA, Panjwani P, Kini U, Mohanty S, Das K. Segmental dilatation of sigmoid colon in a neonate: atypical presentation and histology. J Pediatr Surg.

[10] ( 1989). Martínez MA, Conde J, Bardaji C, Guarch R, Gasco M, Bento L. Congenital segmental dilatation of the colon. Cir Pediatr.

[11] ( 2009). Wakhlu A, Wakhlu AK. Technique and long-term results of coloplasty for congenital short colon. Pediatr Surg Int.

[12] ( 2005). Kothari P, Gowrishankar, Rastogi A , Dipali R, Kulkarni B. Congenital segmental dilatation of colon with colonic atresia. Indian J Gastroenterol.

